# Effects of a single dose of amisulpride on functional brain changes during reward- and motivation-related processing using task-based fMRI in healthy subjects and patients with major depressive disorder — study protocol for a randomized clinical trial

**DOI:** 10.1186/s13063-023-07788-x

**Published:** 2023-11-27

**Authors:** Luisa Carstens, Margot Popp, Christian Keicher, Rita Hertrampf, David Weigner, Marvin S. Meiering, Gerd Luippold, Sigurd D. Süssmuth, Christian F. Beckmann, Andreas Wunder, Simone Grimm

**Affiliations:** 1 MSB Medical School Berlin, Berlin, Germany; 2 Translational Medicine and Clinical Pharmacology, Boehringer Ingelheim Pharma GmbH & Co. KG, Biberach an Der Riss, Germany; 3 Charité Research Organisation GmbH, Berlin, Germany; 4 Clinical Development and Operations, Boehringer Ingelheim Pharma GmbH & Co. KG, Biberach an Der Riss, Germany; 5 Medicine Therapeutic Area CNS-Retinopathies-Emerging Areas, Boehringer Ingelheim International GmbH, Biberach an Der Riss, Germany; 6 Donders Institute, Centre for Medical Neuroscience, Radboud University Medical Centre, Nijmegen, Netherlands; 7 Centre for Functional MRI of the Brain (FMRIB), Nuffield Department of Clinical Neurosciences, Wellcome Centre for Integrative Neuroimaging, University of Oxford, Oxford, UK; 8 SBGneuro Ltd, Littlemore, Oxford, UK

**Keywords:** Anhedonia, Major depressive disorder, fMRI, Amisulpride, Monetary incentive delay task, Instrumental learning task, Effort-based decision-making task, Probabilistic reward task, Social incentive delay task, Resting state

## Abstract

**Background:**

Anhedonia and other deficits in reward- and motivation-related processing in psychiatric patients, including patients with major depressive disorder (MDD), represent a high unmet medical need. Neurobiologically, these deficits in MDD patients are mainly associated with low dopamine function in a frontostriatal network. In this study, alterations in brain activation changes during reward processing and at rest in MDD patients compared with healthy subjects are explored and the effects of a single low dose of the dopamine D2 receptor antagonist amisulpride are investigated.

**Methods:**

This is a randomized, controlled, double-blind, single-dose, single-center parallel-group clinical trial to assess the effects of a single dose of amisulpride (100 mg) on blood-oxygenation-level-dependent (BOLD) responses during reward- and motivation-related processing in healthy subjects (*n* = 60) and MDD patients (*n* = 60). Using functional magnetic resonance imaging (fMRI), BOLD responses are assessed during the monetary incentive delay (MID) task (primary outcome). Exploratory outcomes include BOLD responses and behavioral measures during the MID task, instrumental learning task, effort-based decision-making task, social incentive delay task, and probabilistic reward task as well as changes in resting state functional connectivity and cerebral blood flow.

**Discussion:**

This study broadly covers all aspects of reward- and motivation-related processing as categorized by the National Institute of Mental Health Research Domain Criteria and is thereby an important step towards precision psychiatry. Results regarding the immediate effects of a dopaminergic drug on deficits in reward- and motivation-related processing not only have the potential to significantly broaden our understanding of underlying neurobiological processes but might eventually also pave the way for new treatment options.

**Trial registration:**

ClinicalTrials.gov NCT05347199. April 12, 2022.

**Supplementary Information:**

The online version contains supplementary material available at 10.1186/s13063-023-07788-x.

## Administrative information

**Table:** 

Title {1}	Effects of a single dose of amisulpride on functional brain changes during reward- and motivation-related processing using task-based fMRI in healthy subjects and patients with major depressive disorder — study protocol for a randomized clinical trial
Trial registration {2a and 2b}	A clinical trials registration is available on ClinicalTrials.gov (NCT number: NCT05347199)
Protocol version {3}	3.0; 01 August 2022
Funding {4}	This work is funded by Boehringer Ingelheim Pharma GmbH & Co. KG
Author details {5a}	Luisa Carstens, MSB Medical School Berlin, Germany Margot Popp, Translational Medicine and Clinical Pharmacology, Boehringer Ingelheim Pharma GmbH & Co. KG, Biberach an der Riss, Germany Christian Keicher, Charité Research Organisation GmbH, Berlin, Germany Rita Hertrampf, Charité Research Organisation GmbH, Berlin, Germany David Weigner, MSB Medical School Berlin GmbH, Germany Marvin S. Meiering, MSB Medical School Berlin GmbH, Germany Gerd Luippold, Clinical Development and Operations, Boehringer Ingelheim Pharma GmbH & Co. KG, Biberach an der Riss, Germany Sigurd D. Süssmuth, Medicine Therapeutic Area CNS-Retinopathies-Emerging Areas, Boehringer Ingelheim International GmbH, Biberach an der Riss, Germany Christian F. Beckmann, Donders Institute, Centre for Medical Neuroscience, Radboud University Medical Centre Nijmegen, Netherlands; Centre for Functional MRI of the Brain (FMRIB), Nuffield Department of Clinical Neurosciences, Wellcome Centre for Integrative Neuroimaging, University of Oxford, Oxford, UK; SBGneuro Ltd, Littlemore, Oxford, UK Andreas Wunder, Translational Medicine and Clinical Pharmacology, Boehringer Ingelheim Pharma GmbH & Co. KG, Biberach an der Riss, Germany Simone Grimm, MSB Medical School Berlin GmbH, Germany
Name and contact information for the trial sponsor {5b}	Simone Grimm MSB Medical School Berlin simone.grimm@medicalschool-berlin.de
Role of sponsor {5c}	Responsibility for study design; management, analysis and interpretation of data; writing of the report; and the decision to submit the report for publication. Sponsor has ultimate authority over all activities related to the study
Contact for scientific queries	Simone Grimm MSB Medical School Berlin simone.grimm@medicalschool-berlin.de
Public title	Investigation of the effects of a single dose of amisulpride on reward-associated brain activation

## Introduction

### Background and rationale

Major depressive disorder (MDD) is a leading cause of disability with a worldwide prevalence of over 264 million [1, 2] and represents a high burden for affected individuals, their families, and the society as it comes along with high treatment costs and long periods of hospitalization [2, 3]. Core symptoms are depressed mood and the inability to experience pleasure (anhedonia) [4]. First-line antidepressant treatment approaches often do not relieve anhedonic symptoms [5–7], which constitute a key predictor for poor treatment response [8, 9]. Yet, no dedicated treatment options for anhedonia are available for patients.

One reason why antidepressants have limited efficacy in treating anhedonia is that other than as indicated in the diagnostic criteria, anhedonia does not only encompass reduced capability to feel pleasure. It rather comprises multiple processes and stages related to reward and motivation with different neurobiological correlates involved. A research framework for the investigation of these processes is provided by the National Institute of Mental Health Research Domain Criteria (RDoC) [10], where they are conceptualized in the domain *Positive Valence Systems* comprising the constructs Reward Responsiveness, Reward Learning, and Reward Valuation. The RDoC also provides information about the neurobiological underpinnings of these processes and recommendations regarding tasks and self-report tools for their investigation.

The neurobiological substrates include ventromedial prefrontal/orbitofrontal cortex (vmPFC/OFC), anterior cingulate cortex (ACC), striatum, and ventral tegmental area (VTA) [11, 12] and require dopaminergic signaling [13, 14]. The monetary incentive delay (MID) task is a robust and well-validated task to investigate neural correlates of Reward Responsiveness in the ventral striatum, ACC, and salience network during reward anticipation and reward outcome [15–17]. Commonly used tasks to investigate Reward Learning and Reward Valuation are instrumental and reversal learning tasks, probabilistic reward task, and effort-based valuation tasks [18–21].

Most human studies investigated reward- and motivation-related processing in human experimental settings using monetary reward. However, as other entities like food or social interaction are intrinsically rewarding and therefore comparable according to preclinical research, variations of classical reward tasks also include social reward or food reward [22, 23].

Compared to healthy volunteers (HV), blunted activation in frontostriatal brain regions and poor behavioral responses during reward- and motivation-related decision-making were observed in MDD [24]. Several attempts have been made to investigate the effects of pharmacological intervention on reward- and motivation-related deficits in MDD [25–29]. However, mixed effects have been reported in this context and neurobiological mechanisms underlying deficits in reward- and motivation-related processing are still unclear.

A promising option to investigate treatment effects on reward- and motivation-related deficits is to specifically target dopaminergic neurotransmission in frontostriatal brain regions by using pharmacological interventions that are specific for and have high affinity to dopamine receptors such as amisulpride [25, 29]. Amisulpride’s pharmacological efficacy relates to its high mesolimbic affinity and its properties as a selective dopamine D2/D3 receptor antagonist at the postsynapses at high doses (400–1200 mg per day) [30, 31]. At a low dose (50–300 mg per day), amisulpride has a higher affinity to block presynaptic autoreceptors and thereby increases dopaminergic signaling at the synapse [31].

Amisulpride reduced striatal activation and worsened behavioral performance during an instrumental learning task in HV at a high single dose (400 mg) [32], while at a single lower dose (200 mg) it improved reward learning and enhanced striatal activation during the task [33]. During reward anticipation as assessed using the MID task; however, no effect of a single low dose (200 mg) of amisulpride was observed, which could possibly be due to ceiling effects in healthy subjects [34]. In MDD, associated with dopamine deficiency, single low-dose amisulpride (50 mg) enhanced striatal activations and enhanced connectivity between the ventral striatum and midcingulate cortex during the MID task compared to HV, but did not impact impaired reward learning in MDD patients [29, 35]. The effects of amisulpride on reward valuation in MDD have not been investigated yet.

The aim of this study is to selectively manipulate striatal dopamine signaling using amisulpride during the processing of different reward- and motivation-related tasks. To our knowledge, no functional magnetic resonance imaging (fMRI) study has yet tested in both HV and MDD patients if transiently enhancing dopamine signaling by using a single low dose of amisulpride impacts (1) processing of different reward- and motivation-related tasks that cover all three RDoC constructs of Positive Valence Systems; (2) processing of different reward types (monetary reward and social reward); and (3) functional connectivity at rest and cerebral blood flow.

## Methods

### Study design

This single-center, double-blind, placebo-controlled, randomized, single-dose, parallel-group study is designed to investigate the effects of a single dose of amisulpride on functional brain changes during reward- and motivation-related processing and at rest. The study is conducted by the Charité Research Organisation, a contract research organization and subsidiary of the Charité – Universitätsmedizin Berlin. A total of 120 participants will be recruited. Measurement of functional brain changes is performed after a single dose of amisulpride or placebo in HV and patients with MDD.

It is hypothesized that functional brain changes previously linked to reward- and motivation-related processing require dopaminergic signaling and are diminished in MDD compared to HV. In MDD, but not in HV, the administration of a single low dose (100 mg) of amisulpride should increase brain activation associated with reward- and motivation-related processing.

MDD patients (*n* = 60) and HV (*n* = 60) are randomly allocated in a 1:1 ratio to the two treatment arms (MDD/amisulpride *n* = 30, MDD/placebo *n* = 30, HV/amisulpride *n* = 30, HV/placebo *n* = 30). The effects of a single dose of amisulpride (100 mg) on functional brain changes are assessed during reward- and motivation-related processing and at rest.

The study is composed of four outpatient visits (see Table 1): screening, baseline, and two scanning sessions (visit 3 and visit 4). During screening, subjects provide written informed consent, and all subjects must satisfy all inclusion and exclusion criteria (see eligibility criteria). Further assessments during screening comprise demographics and medical history, physical examination, height and weight, urine pregnancy test, follicle-stimulation hormone (FSH) test, urine drug screen, alcohol breath test, vital signs, electrocardiography (ECG), adverse events, concomitant medication, hematology, chemistry, and coagulation, severe acute respiratory syndrome coronavirus type 2 (SARS-CoV-2) test, as well as the Structured Clinical Interview for DSM-5 (SCID-5) [36], the Montgomery-Åsberg Depression Rating Scale (MADRS) [37], and the Columbia Suicide Severity Rating Scale (C-SSRS) [38]. During the baseline visit, examinations comprise inclusion and exclusion criteria, urine pregnancy test, urine drug screen, alcohol breath test, concomitant medication, and hematology, chemistry, and coagulation. All eligible subjects undergo MRI scanning at visit 3 and visit 4 which are eight days apart from each other. On visits 3 and 4, before the administration of amisulpride or matching placebo, inclusion and exclusion criteria and, again, urine pregnancy test, urine drug screen, alcohol breath test, vital signs, 12-lead ECG, and a SARS-CoV-2 test are assessed. Hematology, Chemistry, and coagulation are examined before drug administration on visit 3 and after drug administration on visit 4. Adverse events and concomitant medication are assessed throughout visits 3 and 4. In the time window between predose and the MRI assessments, all subjects complete several psychopathology questionnaires (Beck Depression Inventory (BDI) [39], Snaith-Hamilton-Pleasure-Scale (SHAPS) [40], Dimensional Anhedonia Rating Scale (DARS) [41], and Leuven Affect and Pleasure Scale (LAPS) [42]) to probe depressive symptomatology and anhedonia.

**Table 1:** Schedule of assessments

Event and comment	SCR	Baseline	Treatment day							Treatment day						
Visit number	1	2	3							4						
Study day^a^	− 28 to − 4	− 3 to − 1	1 2							8 ± 2						
Hours post amisulpride or placebo			Predose	0:00	1:00	1:15	3:30	4:30	4:45	Predose	0:00	1:00	1:15	3:30	4:30	4:45
Written informed consent	**x**															
Inclusion/exclusion criteria	**x**	**x**	**x**							**x**						
Demographics and medical history	**x**															
Physical examination	**x**															**x**
Weight/height	**x**															
Urine pregnancy test^b^	**x**	**x**	**x**							**x**						
FSH test	**x**															
Urine drug screen	**x**	**x**	**x**							**x**						
Alcohol breath test	**x**	**x**	**x**							**x**						
Vital signs^c^	**x**		**x**							**x**						
12-lead ECG	**x**		**x**													**x**
Adverse events	**x**	**x**	**x**							**x**						
Concomitant medication	**x**	**x**	**x**							**x**						
Hematology, chemistry, coagulation^d^	**x**		**x**													**x**
COVID-19 test^e^	**x**	**x**	**x**							**x**						
Randomization		**x**														
Intake of amisulpride or placebo				**x**							**x**					
Breakfast^f^						**x**							**x**			
PK blood amisulpride^g^			**x**		**x**		**x**	**x**		**x**		**x**		**x**	**x**	
Headache severity NRS							**x**	**x**						**x**	**x**	
SCID-5-CV^h^	**x**															
MADRS^i^	**x**															
Columbia Suicide Severity Rating Scale^j^	**x**															
Questionnaires Psychopathology Set^k^				**x**	x	x					**x**	x	x			
MR Scanner trial activity Set 1^l^^((1), (2), (3), (4))^							**x**									
MR Scanner trial activity Set 2^l^^((1), (5), (6))^														**x**		
Behavioral paradigm^m^									**x**							

^a^There is a treatment-free interval of 7 ± 2 days between visits 3 and 4

^b^Urine pregnancy test (if urine test positive, blood test to be done)

^c^Vital signs: pulse rate, systolic and diastolic blood pressure (BP), and body temperature in the ear

^d^Hematology: leukocytes, granulocytes, neutrophils, eosinophils, basophiles, lymphocytes, monocytes, erythrocytes, thrombocytes, hematocrit, hemoglobin; chemistry: sodium, potassium, calcium, magnesium, total protein, albumin, glucose, creatinine, urea, bilirubin, ASAT, ALT, GGT, LDH, AP, CRP; Coagulation: aPTT, INR; * no coagulation at Visit 3 and 4

^e^Prior to Randomization

^f^Time window: 1:15 ± 15 min after amisulpride intake

^g^Time windows: 1:00 + 5 min, 3:30 + 30 min, 4:30 + 30 min

^h^SCID-5-CV (DSM-5, Beesdo-Baum, Zaudig, and Wittchen, 2019); * within 14 days prior to visit 3

^i^MADRS (Montgomery-Åsberg Depression Rating Scale, Montgomery and Åsberg, 1979); * within 14 days prior to visit 3

^g^Columbia Suicide Severity Rating Scale (C-SSRS, Posner et al., 2011); * within 14 days prior to visit 3

^k^Questionnaires Set: Beck Depression Inventory (BDI; Beck et al., 1961); Snaith-Hamilton-Pleasure-Scale (SHAPS; Snaith et al., 1995); Dimensional Anhedonia Rating Scale (DARS; Rizvi et al., 2015); Leuven Affect and Pleasure Scale (LAPS; Demyttenaere et al., 2019)

^l^MRI: (1) high-resolution anatomical MRI scan; (2) resting-state functional magnetic resonance imaging (rsfMRI); (3) MID task; (4): instrumental learning fMRI task; (5) SID task; (6) effort-based decision-making fMRI task; (7) arterial spin labeling (ASL)

^m^Time window for probabilistic reward task: ± 30 min

**Fig. 1:**
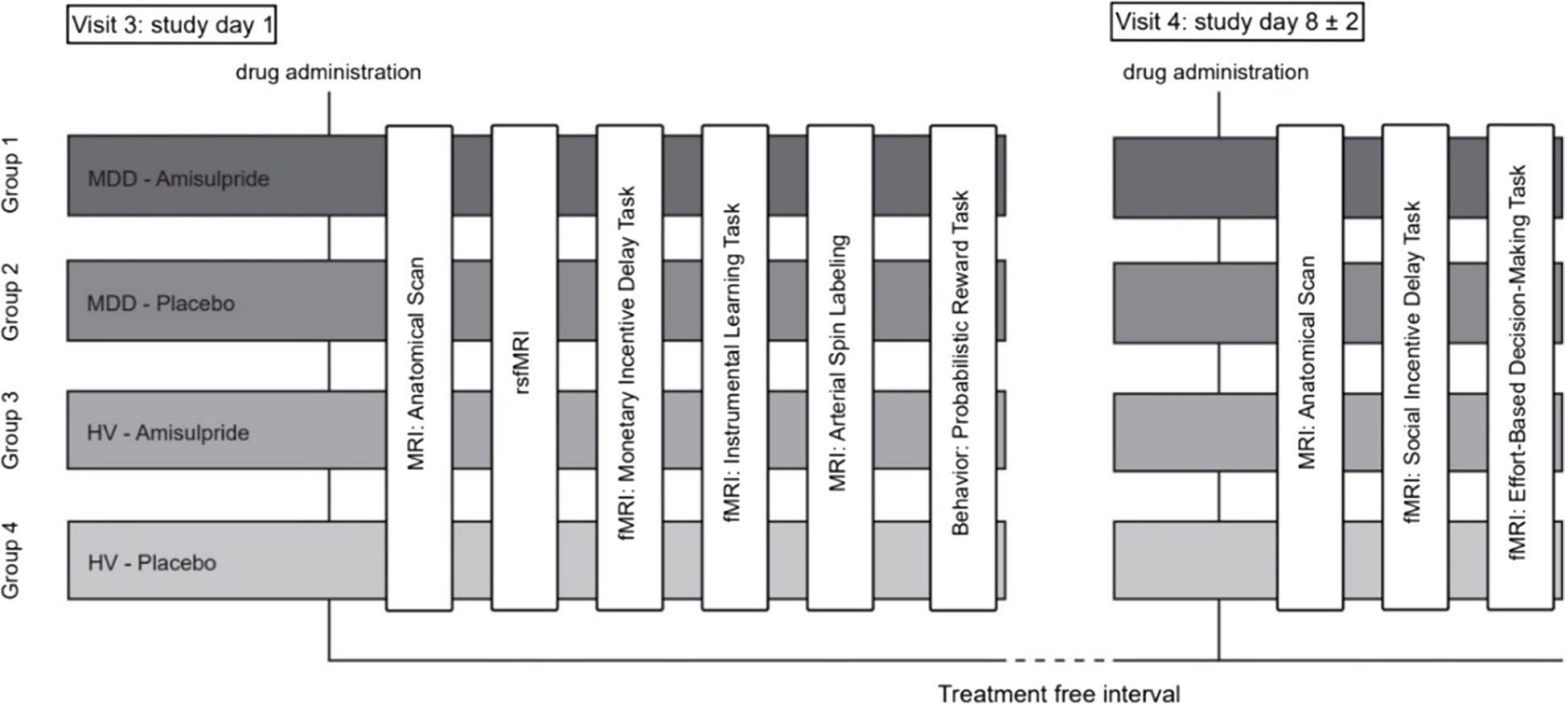
Timeline for substance administration and MRI protocol for each experimental group at visit 3 and visit 4

Treatment with amisulpride or matching placebo occurs 3.5 to 4 h before the start of each scanning session. 1 to 1.5 h after the intake of amisulpride or matching placebo, subjects are served a standardized breakfast. Multimodal MR brain imaging (high-resolution anatomical MRI scan, resting state fMRI, task-based fMRI, arterial spin labeling (ASL)) is performed on two separate days where subjects first undergo an anatomical scan. On visit 3, the anatomical scan is followed by a resting state scan, two fMRI tasks (MID task [15] task and instrumental learning task (ILT) [21], an ASL sequence, and the probabilistic reward task (PRT) [18] as a pure behavioral measure of reward learning which is performed outside the scanner. The MID task is the first task presented inside the scanner to prevent it from being affected by fatigue as it is the primary outcome. On visit 4, the anatomical scan is followed by two different fMRI tasks (social incentive delay task (SID) [43] and effort-based decision-making (EBDM) task [20]. The SID task is presented before the EBDM task as physical effort is required to perform the assessments. Headache severity is measured using a numeric ranking scale to control for possible confounding of the MRI assessments by headache. MRI assessments are conducted at two visits to not exceed a scanning time of 60 min each. A timeline for substance administration and MR protocol for each experimental group at visit 3 and visit 4 can be found in Fig. 1. Time of treatment is standardized across subjects.

At visit 3 and visit 4, blood samples are taken 30 min pre-dose, and 1 h, 3.5 to 4 h, and 4.5 to 5 h after oral drug administration to assess the pharmacokinetics of amisulpride. On visit 4, after the MRI measurements, a physical examination, as well as 12-lead ECG, hematology, chemistry, and coagulation, is performed. A description and a detailed schedule of assessments can be found in Table 1, including questionnaires, laboratory tests, MRI, and the behavioral paradigm.

All assessments are performed by trained professionals in a standardized manner. In order to ensure high-quality standards of the assessments, a common digital manual as well as quality monitoring procedures are implemented (see the “Data management and monitoring” section).

This study was designed by the MSB Medical School Berlin (LC, DW, MSM, SG) and Boehringer Ingelheim Pharma GmbH & Co. KG (MP, GL, SDS, AW). The Charité Research Organisation (CK, RH), a contract research organization and subsidiary of the Charité – Universitätsmedizin Berlin, is responsible for the implementation and, together with the MSB Medical School Berlin, conduction of the trial. SG (MSB Medical School Berlin) is the grant holder and sponsor. SBGneuro Ltd (CB) is responsible for the statistical analysis plan including power analysis. For the interpretation of the data, the responsibility lies with the researchers of the MSB Medical School Berlin. Study organization, recruitment of participants, and monitoring are performed by the Charité Research Organisation (CK, RH). This study is conducted in compliance with Good Clinical Practices (ICH-GCP) and the Declaration of Helsinki. A clinical trial registration is available on ClinicalTrials.gov (NCT number: NCT05347199). The trial is funded by Boehringer Ingelheim Pharma GmbH & Co. KG and approved by the respective local ethics committee (Landesamt für Gesundheit und Soziales, Geschäftsstelle der Ethik-Kommission des Landes Berlin, Berlin, Germany; reference number: 22/029 – IV E 11) and the Bundesinstitut für Arzneimittel und Medizinprodukte, Bonn, Germany.

The reporting of this study protocol was performed according to the Standard Protocol Items Recommendations for Interventional trials (SPIRIT; [44]).

### Eligibility criteria

The main inclusion criteria for all subjects are.

- Male or female
- Aged 18 to 45 years

The main exclusion criteria for all subjects are:

- Contraindications to MRI conduction
- Contraindication to amisulpride intake according to label information
- QT prolongation in ECG
- A positive SARS-CoV-2 test.
- Having received prescribed medication within 14 days prior to visit 3 (apart from the contraceptive pill)

The main inclusion criteria for the MDD group are:

- Patients with MDD
- A MADRS score ≥ 7 and < 26 at screening

The main exclusion criteria for the MDD group are:

- Meeting diagnostic criteria for any major psychiatric disorder (other than MDD), as determined by SCID-5 at screening
- Having received fluoxetine within 90 days prior to visit 3
- Having received psychotherapy within 14 days prior to visit 3

The main inclusion criteria for the HV group are:

- Healthy

The main exclusion criteria for the HV group are:

- Meeting diagnostic criteria for any major psychiatric disorder, as determined by SCID-5 at screening
- A lifetime history of psychiatric or neurologic disorders

### Recruitment of participants and informed consent

All study participants are recruited by the clinical research unit (Charité Research Organisation). It is anticipated that approximately six to eight subjects will be enrolled per month. The anticipated enrolment period is from June 2022 until September 2023.

Prior to performing any study-related procedure at screening, each potential subject must provide a signed and dated acknowledgement of their freely given informed consent (informed consent form (ICF)). Either the investigator or a designated person explains the aims, methods, anticipated benefits, and potential hazards of this protocol and any discomfort it may entail, and the subject is given an opportunity to discuss any questions regarding the trial procedures and conduction. The subject is allowed sufficient time to consider trial participation. The subject receives a signed copy of the ICF.

### Study discontinuation

Subjects can withdraw from the study at any time without consequences and without giving a reason. Subjects who choose to withdraw from the study or are withdrawn by the investigator (i.e., in case of adverse events or protocol violations) are defined as early study discontinuation. The date of discontinuation must be documented in the subject file and in the electronic case report form (eCRF) and sponsor and, if applicable (i.e., if the principal investigator (PI) is already aware of the dropout because of the PI being in direct contact with the subject, it is not needed to inform the PI in written form), the PI must be informed in written form by the study team. The PI can also discontinue the study if new safety concerns arise which are considered of relevant change to the benefit/risk assessment. The date of and the primary reason for the withdrawal, as well as the observations available at the time of withdrawal, are to be documented in the eCRF.

Reasons leading to the withdrawal of a subject can include the following (one primary reason must be determined):

- Intolerable adverse events
- Study Stopping Rules (see next section)
- Lack of subject’s cooperation, e.g., subject’s request to withdraw, lack of compliance
- Other reasons (noting reason), e.g., important protocol deviations such as in/ex criteria not met but discovered only after randomization

In all subjects who finish the study prematurely, an early discontinuation examination, if possible, should be carried out.

### Study stopping rules

The study may be terminated at any time for any reason by the sponsor. The entire trial must be terminated prematurely including, but not limited to, the following reasons:

- The sponsor or the investigator considers that the number and/or severity of adverse events justify the discontinuation of the study
- New information is received regarding product safety or other issues arise related to the trial that precludes its completion (e.g., manufacturing issues such as unavailability of amisulpride or a placebo pill, insufficient overall recruitment of subjects, economic reasons such as the insufficient budget for the trial or insolvency of the sponsor or the Charité Research Organisation).

The reason for such a decision will be documented in writing. The respective independent ethics committee (IEC) and the applicable competent authority will be informed in writing as appropriate.

In case one, related (= causal relationship with the trial drug cannot be excluded) serious adverse events or repeated (≥ 2) severe related adverse events are reported, the trial will be interrupted, and no further treatments will be administered. The trial will only be restarted after a substantial amendment of the trial application has been submitted by the sponsor and a favorable opinion of the health authority and the ethics committee has been received. Subjects that already received treatment will be followed further to collect safety data until all adverse events possibly related to study participation or any study-associated procedure are followed up until they have resolved, have been sufficiently characterized or no further information can be obtained.

### Relevant concomitant care

During the screening period, prior and concomitant medications are recorded and reviewed throughout the study until visit 4. In particular, prescribed medication or psychotherapy as mentioned above are not permitted as concomitant treatments. Subjects who have taken prescription or non-prescription medication may still be entered into the study, if, in the opinion of the investigator, the medication does not interfere with the study procedures or compromise safety. Amisulpride will not be used in combination with medications which, e.g., could induce torsade de pointes (such as class Ia and class III antiarrhythmic agents), as well as other medications as indicated in the label of amisulpride.

If concomitant drugs are administered, these are recorded in the subjects’ file and eCRF. Permitted concomitant treatments are not to be changed during the course of this study.

Moreover, the following criteria concerning diet and lifestyle result in the withdrawal of the study subject:

- Consumption of large amounts of caffeinated drinks (more than 8 cups of standard caffeinated drinks (tea, instant coffee) or 6 cups of stronger coffee or other drinks containing methylxanthines such as Coca-Cola or Red Bull per day
- Consumption of caffeinated drinks 2 h prior to the start of the fMRI scan
- Smoking/nicotine consumption 2 h prior to the start of the fMRI scan
- Consumption of food containing poppy seeds within 24 h prior to visits 3 and 4
- Consumption of alcohol within 24 h prior to visits 3 and 4.

As this study enrolls also women who are considered to be of childbearing potential, suitable measures must be taken to avoid falling pregnant. Male participants and female participants of childbearing potential must maintain highly effective contraception methods starting from the signing of the informed consent form and for at least 3 months after the last dose of study treatment. Men must agree to use condoms (including men who have had vasectomies) even if their partner is pregnant (this is to ensure that the fetus is not exposed to the study drug through vaginal absorption) and to not donate sperm and to not plan to father a child during the study and for 3 months after receiving the last dose of study drug. Male subjects must be counseled by the investigator that they should encourage their female partner to use a highly effective method of contraception consistent with local regulations regarding the use of contraceptive methods for subjects participating in clinical studies (e.g., prescription oral contraceptives, contraceptive injections, intrauterine device (IUD)) in addition to the condom used by the male study subject. Rescreening of withdrawn subjects is allowed once at the discretion of the investigator.

### Group randomization

After written informed consent is obtained, healthy and MDD subjects meeting all in-/exclusion criteria are randomized at baseline (visit 2) or visit 3 to receive either amisulpride or matching placebo.

Treatments are randomly allocated in a 1:1 ratio. Randomization is performed in accordance with prepared randomization lists provided by an independent vendor of the Charité Research Organisation. A qualified external pharmacy performs a patient/subject individual re-packaging of the study medication in capsule boxes in accordance with the unblinded randomization list. Each capsule box is labeled with the respective randomization number and contains the study medication (amisulpride or matching placebo). The label does not allow any inferences about the administered compound.

The randomization list is kept in safe and confidential custody. Only personnel not involved in study conduction has access to the list. Additionally, sealed emergency envelopes containing information on the subject’s trial medication are provided to the Charité Research Organisation.

Thereupon, the unblinded Study Team of the Charité Research Organisation receives one capsule box with the corresponding tablet (amisulpride or matching placebo) for each patient/subject per visit. The blinded investigator administers two single doses of the same blinded study medication (amisulpride or matching placebo) to each subject according to the respective randomization number.

Additional randomization numbers are used in case of replacements being needed. The investigator enrolls study participants and documents the randomization number in the eCRF.

### Pharmacological intervention

Amisulpride is an atypical antipsychotic of the benzamine class. It is a well-tolerated selective dopamine D2/3 receptor antagonist with high mesolimbic affinity [30, 31]. In Germany, amisulpride is approved for the treatment of positive and negative symptoms in acute and chronic schizophrenic disorders.

Amisulpride expresses dose-dependent effects as shown in preclinical research. At a high dose, amisulpride reduces dopaminergic neurotransmission at the postsynapses by antagonizing D2/D3 receptors. At a low dose, however, amisulpride has a higher affinity for presynaptic autoreceptors and thereby enhances dopamine signaling [30, 31]. In this study, two single low doses of amisulpride (100 mg) or matching placebo are administered. Since the aim of this study is to explore the the effects of modified dopaminergic signaling on reward circuits in the brain rather than the antidepressant effect of amisulpride, a placebo will be used in the control group instead of a standard antidepressant treatment.

Amisulpride is a commercially available product and the standard dose to treat negative symptoms in schizophrenia is 50–300 mg [31]. Hence, the administered dose in this study (100 mg) lies well within this range. Previous trials investigating the effects of low-dose amisulpride on reward- and motivation-related processing using fMRI reported no serious adverse events [29, 33, 35]. Amisulpride is well tolerated at low doses and has no clinically significant effects on memory and psychomotor performance [31, 45].

In this study, amisulpride and matching placebo are administered orally in the form of a white tablet under fasted conditions 3.5 to 4 h before the start of fMRI scanning at both Visit 3 and Visit 4 to ensure maximum plasma levels during the tasks. The placebo tablet was manufactured by Zentiva Pharma GmbH to resemble the amisulpride tablet. Medication is administered by the blinded site staff of the Charité Research Organisation. None of the investigators, neither the attending physician nor the study team of MSB Medical School Berlin know which treatment the respective subject received.

Plasma samples are collected to determine the blood levels of amisulpride. Blood samples are taken at pre-dose, and 1 h, 3.5 to 4 h, and 4.5 to 5 h after oral drug administration to determine pharmacokinetic parameters of amisulpride. Drug supplies are kept in their original packaging and in a secure limited access storage area according to the recommended storage conditions on the medication label. A temperature log is maintained for documentation. A standardized breakfast is served to subjects after administration of blinded study medication. Daytime of treatment is standardized across subjects. No drug dose changes will be performed.

### Strategies to improve adherence to interventions

All study participants receive medical monitoring and counseling during study participation. In addition, should they wish, participants are informed about the results of their task performance after the finalization of the study. By scheduling the study participants, it is guaranteed that the waiting time is short.

Study participants receive a financial remuneration, which covers all costs incurred by study participation. The financial remuneration is the same for HV and for MDD patients.

### Provisions for post-trial care

Adverse events are recorded at each site visit. Adverse events possibly related to study participation, or any study-associated procedure are followed up until they have resolved, have been sufficiently characterized or no further information can be obtained.

In the case that a study participant dies, or the body or health of the study participant is injured due to the participation in the clinical trial, there is an insurance policy in accordance with Sect. 40, paragraph 1, sentence 3, no. 8 of the German Medicines Act (AMG), which provides benefits even if no one else is liable for the damage.

### Endpoints

The primary endpoint of efficacy is:

- Blood-oxygenation-level-dependent (BOLD) fMRI parameter estimates (ß-weights within the generalized linear model (GLM) analysis) will be extracted from task-related regions of interest (ROI) (average %BOLD signal change and 90th percentile thereof within ROI) under the following task-specific contrast
- MID task:
  - ◦ Contrast of “high-gain” vs. “no-gain” condition during the task cue period
- ROI:
  - ◦ Ventral striatum (including nucleus accumbens)

Exploratory endpoints of efficacy are:

- BOLD fMRI parameter estimates (ß-weights within the GLM analysis) will be extracted from task-related regions of interest (average %BOLD signal change and 90th percentile thereof within ROIs) under the following task-specific contrasts:
  - SID task:
    - ◦ Contrast of “high-gain” vs. “no-gain” condition during the task CUE period
  - ILT:
    - ◦ Contrast of the “gain-cue” vs. “neutral cue” conditions during the task cue and feedback periods
    EBDM task:
    - ◦ Contrast of the “high reward” vs. “low reward” conditions during the task cue2 period
    - ◦ Contrast of the “high effort” vs. “low effort” conditions during the task Cue2 period
  - ROIs for all tasks:
    - ◦ Ventral striatum (including nucleus accumbens), VTA, dorsal ACC, insula, vmPFC/OFC, and ventral pallidum
- Reaction times and estimates of response accuracy extracted from the in-scanner protocol log files
- BOLD fMRI signal magnitude and BOLD signal standard deviation during Resting State within the following a priori defined regions:
  - ◦ Default Mode Network (posterior cingulate, vmPFC, and medial temporal lobe), Central Executive Network (dorsolateral PFC, premotor cortex, precuneus), and Salience Network (amygdala, insula, and dorsal ACC)
- Increases in relative and absolute cerebral blood flow measured through arterial spin labeling MR in the whole brain and in the following brain regions:
  - ◦ (Bilateral): ventral striatum, vmPFC/PFC, VTA, dorsal ACC, insula, and ventral pallidum after amisulpride administration<div class="NodiCopyInline">◦ </div>
- Changes in associations between the magnitude of BOLD signal during reward-and motivation-related processing, self-reported anhedonia, and behavioral measures after amisulpride administration as compared to placebo in MDD patients relative to HV
- Changes in associations between resting state functional connectivity, self-reported anhedonia, and behavioral measures after amisulpride administration as compared to placebo in MDD patients relative to HV
- Changes in plasma levels of amisulpride including correlation to changes in BOLD signal in MDD patients relative to HV
- Changes in whole brain BOLD signal after amisulpride administration as compared to placebo in MDD patients relative to HV

### Statistical analysis

All fMRI data (pre)processing and analysis are performed using FSL (Oxford, UK) according to custom practice and pipelines using the batch or Python programming languages. Additional data analyses are performed using SPSS software (IBM SPSS, New York, USA). Descriptive statistics (mean ± SD) are presented for all primary and exploratory outcomes. The statistical model to compare the primary and exploratory outcomes between the four groups MDD/placebo, MDD/amisulpride, HV/placebo, and HV/ amisulpride is an analysis of variance (ANOVA) including the main effects of treatment (amisulpride/placebo), group (MDD/HV) as well as interaction effects. Appropriate effect sizes (e.g., Cohen’s *d*) are reported. A detailed description of all statistical procedures for the fMRI analyses can be found in the statistical analysis plan in the appendix.

### Sample size

Power analysis for a one-way ANOVA with 4 groups was conducted in G*Power [46] to determine a sufficient sample size using a one-sided alpha of 0.05, a power of 0.95, and a large effect size (*d* = 0.84). Previous studies in HV and in patients with MDD found large effects (*d* > 0.8), showing a significant increase in striatal activity after amisulpride administration as compared to placebo in patients with MDD [32, 35]. The sample size needed with this effect size is *N* = 102 completers. In order to have the same number of subjects per group, we aim for 104 completers, i.e., 26 per group. Accounting for a drop-out rate of ∼15%, the estimated total sample size is *N* = 120 (30 subjects per group).

Screening for the trial will stop once a sufficient number of subjects has been measured and controlled for dropouts due to excessive head movement in the scanner and/or headaches and incomplete data.

### Protocol deviations and handling of missing values

Data from subjects who prematurely terminate the trial are used up to the maximum extent possible. Subjects who discontinue the trial early before the administration of study drug are replaced from the trial before the administration of the study drug. No procedures for replacing missing data are intended. All protocol non-compliances are listed and the reasons for exclusion of subjects from any of the analysis sets are listed. Where relevant, the data from these subjects is described separately.

### Data management and monitoring

Data is entered by the data entry team of Charité Research Organisation into the eCRF medrio. Instructions for use of the system and completion of the eCRF is outlined in the eCRF completion guideline. Quality assurance reports are generated to ensure study data are clean, accurate, and complete. Quality assurance reports include, but are not limited to, the following: missing forms, missing and out-of-range values, automated data queries, and targeted manual reviews of study data. Charité Research Organisation monitoring staff conduct site visits for source data verification to ensure appropriate quality and completeness of data. Discrepancies in data entry in eCRFs will trigger data re-entry requirements and/or site retraining for the relevant data fields.

For each subject randomized, an eCRF is completed within the Electronic Data Capture (EDC) system and approved by the investigator.

All data entered in the eCRF is verifiable in source documentation other than the eCRF. Source documents are filed at the trial site according to local standard operating procedure (SOP). Trained study site staff is responsible for entering subject source data into the validated EDC system. Data captured electronically is immediately saved to the applicable database and changes are tracked to provide an audit trail. Data validation procedures are applied by Data Management to each stage of data handling to ensure that all data are reliable and have been processed correctly.

Data Management SOPs are in place for EDC Setup, EDC User Management, Data Validation, and Database Lock. The EDC database is locked once all expected data are captured in EDC, all discrepancies are resolved and all medical/surgical terms and medication are classified by the latest versions of Medical and Drug dictionaries.

The medical operations team and the project management team of the Charité Research Organisation organize and perform site training, monitoring, and close oversight of all study visits to ensure proper adherence to the clinical trial protocol and to international research standards. Monitoring visits include regular review of data submission forms, source data verification, informed consent documentation, and adverse event reporting. The sponsor (MSB Medical School Berlin) also monitors compliance with the protocol and Good Clinical Practice.

### Frequency and plans for auditing trial conduct

Monitoring is conducted by regular on-site monitoring visits and inhouse data quality review. The frequency of site monitoring is determined by assessing all characteristics of the trial, including its nature, objective, and methodology.

The investigator/institution allows trial-related site monitoring, audits, institutional review board (IRB)/IEC review, and regulatory inspections. Direct access is provided to the eCRF and all source documents/data, including progress notes and copies of laboratory and medical test results, which are available at all times for review by the clinical research associate, auditor, and regulatory inspector. They may review all eCRFs and informed consent forms. The accuracy of the data can be verified by direct comparison with the source documents. A quality assurance audit/inspection of this trial may be conducted by the sponsor, sponsor’s designees, IRB/IEC, or regulatory authorities. The quality assurance auditor will have access to all medical records, the investigator’s trial-related files and correspondence, and the informed consent documentation of this clinical trial.

### Confidentiality

Medical information obtained for individual subjects during the trial is always considered confidential, and this is ensured by using individual subject identification code numbers.

Data is transferred and shared in pseudonymized form, and all data processing and storage follows general data protection regulations and national law requirements. Further information on confidentiality is provided in the informed consent form.

### Blinding

In this double-blind study, participants as well as investigators and data analysts are blinded. An unblinded site team provides the emergency envelopes using the random list. The investigator receives a set of sealed envelopes, one for each randomization number. These envelopes contain information on the subject’s trial medication and are to be opened by the investigator or an authorized person only under circumstances, in which it is medically imperative to know what the subject is receiving. The randomization envelopes are not to be opened by the investigators at the end of the trial. The investigators or the person who breaks the blind must record the date and the reasons for doing so in the eCRF, in the subject’s medical record, and on the randomization envelope.

In compliance with applicable regulations, in the event of a suspected unexpected serious adverse reaction (SUSAR) related to the blinded treatment, the subject’s treatment code is usually unblinded before reporting to the health authorities. Notifications of the ethic committee and investigators will be done according to all applicable regulations.

### Safety monitoring

Adverse events are interrogated for at each contact between the responsible investigator and the study subject. Furthermore, all pathological and clinically relevant findings in physical examinations, vital signs, 12-lead ECGs, clinical chemistry, hematology, and coagulation are documented as adverse events.

Wherever possible, adverse events are reported on the basis of CTCAE v5.0. Adverse events are reported with subject-ID, start and end date, description, grading, seriousness, relationship, action taken, and outcome.

### Plans for communicating important protocol amendments to relevant parties (e.g., trial participants, ethical committees)

Any significant change in the study requirements, design, or scheduled activities requires a protocol amendment to be issued. The investigator must not make any changes to the study or deviate from this protocol without competent regulatory authority(s), ethical committee, and sponsor approval, except when necessary to avoid immediate danger to subjects. A change in procedures specified in this protocol that is intended to remove an immediate danger to subjects may be implemented immediately. The change must be documented and reported to the ethical committee and the appropriate regulatory authority(s). An appropriate amendment to the protocol is to be implemented. All protocol amendments must be reviewed and approved in the same manner as the protocol.

## Discussion

With this study in healthy subjects and patients with MDD, the effects of a single low dose of amisulpride on functional brain changes during reward- and motivation-related tasks are investigated. By an innovative approach, this study broadly covers all aspects of reward- and motivation-related processes. To the best of our knowledge, no imaging study using pharmacological intervention has implemented assessments to cover all constructs of the RDoC domain Positive Valence Systems before. The study provides a systematic setting to explore, characterize, and disentangle reward- and motivation-related deficits in subjects suffering from MDD compared with healthy subjects at the neural as well as at the behavioral level. By the administration of a selective dopaminergic intervention, drug effects on the involved neural circuits can be investigated for the respective aspects of reward and motivation.

In this study, patients with MDD are included to increase comparability to other studies as major depression is one of the most frequently investigated mental disorders in the field of reward and motivation (for a review, see [24]). Specifically, currently unmedicated patients with mild to moderate MDD are assessed to prevent possible confounding effects of co-medication on study outcomes and to reduce the probability that patients additionally experience psychomotor retardation as this commonly occurs in patients suffering from major depression [47].

Amisulpride was selected as a pharmacological intervention as it specifically influences dopaminergic neurotransmission at D2 and D3 receptor subtypes with low affinity to other receptor types [31]. The mode of action of amisulpride is dose-specific: While high doses (starting from 400 mg) are associated with a reduction of dopaminergic signaling, low doses (300 mg and less) are supposed to enhance dopaminergic signaling in mesolimbic brain regions [31]. There is evidence from both preclinical and clinical studies that a low dose of amisulpride leads to improvement of deficits in reward- and motivation-related processing [29, 48]. In this study, we considered the pharmacokinetic and -dynamic profile of amisulpride published in the literature [31, 49–51] and optimized the dose and the time point of assessments accordingly to achieve an optimal pharmacodynamic effect on the dopamine system. The ideal time window for data collection was derived from the concentration maximum of amisulpride and the pharmacodynamic effect on dopamine signaling in the brain [31, 50]. Potential food effects [52] were considered by providing a standardized breakfast at a fixed timepoint after drug administration to subjects. The time of treatment is standardized across subjects to avoid possible confounding effects of circadian variation of dopamine receptor activity. During the measurement period, a relatively constant dopamine level can be expected in this setting, based on the known pharmacokinetics of amisulpride. Therefore, we assume to have an optimal setting to investigate the effects of a single dose of amisulpride on functional brain changes during reward- and motivation-related processing.

The study is designed for a single-dose administration. For multiple dose studies, the task design would not be optimal, since some of the tasks involve learning processes, which would make repeated measurement unreliable. For the same reason, a parallel group design is preferable over a cross-over design. To avoid exceeding scanning time over 1 h, brain imaging is split into two sessions. The sequence of MRI assessments was carefully selected: the resting-state sequence is performed prior to the fMRI tasks, as resting-state MRI is prone to carry-over effects from previous tasks. ASL is less affected by these carry-over effects and therefore implemented at the end of the first scanning session. Careful consideration was given to the order of fMRI tasks so that fatigue does not affect the primary endpoint. Therefore, the MID task is implemented before the Instrumental Learning task during the first scanning session. At the first scanning day (visit 3), measurements are concluded with the PRT outside the scanner as a pure behavioral measure of Reward Learning. The PRT is presented only during the first scanning visit and will therefore not be affected by dropouts during the second visit. During the second scanning session, the EBDM is performed after the SID task, because the physical effort during this task might otherwise affect the processing of and the performance during the SID task. Finally, the time window between the two scanning days ensures a complete washout of amisulpride by the time the second single dose is administered.

The clinical study protocol was first submitted on February 11, 2022, to the competent authorities. The independent ethics committee approved the protocol on March 3, 2022, with conditions. One condition was that it should be guaranteed that MDD patients enrolled in the trial were diagnosed and treated by staff experienced and certified for the treatment of major depressive disorder (psychiatrists, psychologists). The protocol amendment includes that subjects treated with antidepressants would not be included in the study and that subjects who received antidepressant treatment prior to study enrollment should not interrupt or wash out treatment for study purposes. Furthermore, it was specifically mentioned that no subjects with a QT prolongation at screening ECG should be enrolled in the study. An additional ECG assessment was included predose at Visit 4 for safety reasons. In addition, in the amended clinical study protocol clarifications to the study endpoints, study objectives, contraindicated concomitant medications, maximum number of subject replacements, treatment randomization, replacement in the event of headache, sample size, statistical analysis, and minor grammatical, formatting, or spelling corrections were made. After receiving final approval from the independent ethics committee and the Bundesinstitut für Arzneimittel und Medizinprodukte, recruitment was started.

Screening procedures of the first healthy volunteers revealed that the lower limits for systolic and diastolic blood pressure (100 mmHg and 65 mmHg) needed to fulfill eligibility criteria were defined too narrowly. More than 50% of all screened healthy volunteers could not pass the screening visit successfully. With an amendment of the clinical study protocol, the lower limits of blood pressure were reduced to 50 mmHg for diastolic blood pressure and to 90 mmHg for systolic blood pressure. Since a reflex tachycardia might occur in response to low blood pressure, the upper limit of the pulse rate was increased to 100 bpm (instead of 90 bpm) for inclusion. With the implementation of these new limits (after approval by the independent ethics committee and the Bundesinstitut für Arzneimittel und Medizinprodukte) the screening process was optimized resulting in a reduced screen failure rate.

The authors believe that this study contributes to a better understanding of the underlying neurobiology of reward and motivation-related processes as well as the pathophysiology in patients. Furthermore, the study investigates the effects of enhanced dopaminergic signaling in mesolimbic brain regions induced by amisulpride treatment. Therefore, it might constitute an important step on the way to a more precise treatment of patients suffering from reward- and motivation-related deficits. Results of the study might support the use of the described assessments as tools to assess pharmacodynamic effects in the development of new drugs.

## Trial status

The reported protocol version is 3.0, approved on 1 August 2022. Recruitment was started on 17 May 2022 and is ongoing. The study completion date is estimated to be in September 2023.

### Supplementary Information

**Additional file 1.**

**Additional file 2.**

## Data Availability

The pseudonymized final trial data set will be available to all parties involved in the conduct of this study (Charité Research Organisation, SBGneuro, Boehringer Ingelheim Pharma, MSB Medical School Berlin) under the conditions specified in the confidentiality section above. Furthermore, the final trial dataset will be made publicly available via the G-Node Infrastructure (https://gin.g-node.org/). The statistical code will be available from the corresponding author on reasonable request, as is the full protocol.

## Abbreviations

ACC: Anterior cingulate cortex
AMG: German Medicines Act
ANOVA: Analysis of variance
ASL: Arterial spin labeling
BOLD: Blood-oxygen-level-dependent
CTCAE: Common Terminology Criteria for Adverse Events
EBDM: Effort-based decision-making
ECG: Electrocardiogram
eCRF: Electronic case report form
EDC: Electronic data capture
fMRI: Functional magnetic resonance imaging
FSH: Follicle-stimulation hormone
FSL: FMRI Software Library
GLM: Generalized linear model
HV: Healthy volunteers
ICF: Informed consent form
IEC: Independent ethics committee
ILT: Instrumental learning task
IRB: Institutional review board
MADRS: Montgomery-Åsberg Depression Rating Scale
MDD: Major depressive disorder
MID: Monetary incentive delay
MR: Magnetic resonance
MRI: Magnetic resonance imaging
PFC: Prefrontal cortex
PI: Principal investigator
PRT: Probabilistic reward task
RDoC: Research Domain Criteria
ROI: Region of interest
SARS-CoV-2: Coronavirus type 2
SCID-5: Structured Clinical Interview for DSM-5
SID: Social incentive delay
SOP: Standard operating procedure
SPIRIT: Standard Protocol Items Recommendations for Interventional Trials
SUSAR: Suspected unexpected serious adverse reaction
vmPFC/OFC: Ventromedial prefrontal/orbitofrontal cortex
VTA: Ventral tegmental area
