# Supplementary material for: Effects of a single dose of amisulpride on functional brain changes during reward- and motivation-related processing using task-based fMRI in healthy subjects and patients with major depressive disorder — study protocol for a randomized clinical trial
Source: Trials. 2023 Nov 27;24:761. doi: 10.1186/s13063-023-07788-x (PMC10683198; doi:10.1186/s13063-023-07788-x)
Supplement: Supplementary file 1 — Additional file 1. [file 13063_2023_7788_MOESM1_ESM.pdf]

SBGneuro

# Statistical Analysis Plan

MSB-C002

Version 1.0 - final

Exported on March 23 2022

SBGneuro

# Statistical Analysis Plan

exported on March 23 2022

## Electronic Signature

| | |
|---|---|
| Status | |
| Approver | |
| Approval Date | |
| Expiration Date | |
| Version | 1.0 - final |

## Company Propriety Information

Copyright © 2022 SBGneuro Ltd. All rights reserved.

The material presented in this document and contained herein is proprietary and confidential to SBGneuro Ltd. and is intended for SBGneuro employees and its clients use only.

Material provided herein is confidential property and copyright protected by SBGneuro Limited. No part of said material can be reproduced or transmitted in any form or by any means, electronic or mechanical, including photocopy, recordings, emailing, or any information storage and retrieval system now or to be invented, without permission in written form from:

**SBGneuro Limited**

**John Eccles House**
**Science Park**
**Robert Robinson Ave**

SBGneuro

**Littlemore**
**Oxford, OX4 4GP**
**United Kingdom**

**Registered in England & Wales (No. 08183417)**

# Table of Contents


| | | |
|---|---|---|
| 1 | Document preamble | 5 |
| 2 | Document revision history | 8 |
| 3 | Approval | 10 |
| 4 | Executive Summary | 12 |
| 4.1 | Introduction | 12 |
| 4.2 | Study Design | 12 |
| 4.3 | Trial Objectives and Endpoints | 12 |
| 4.4 | Blinding and Unblinding | 13 |
| 4.5 | Image Acquisition | 13 |
| 4.6 | Endpoints and reporting | 14 |
| 5 | MRI Analysis Design and Methodology | 15 |
| 5.1 | Schedule for MRI Analysis | 15 |
| 5.2 | Data for MRI Analysis | 15 |
| 5.3 | Handling Missing Data | 15 |
| 5.4 | MRI Quality Control | 15 |
| 5.5 | MRI Analysis | 18 |
| 6 | References | 26 |
| 7 | Appendix | 28 |
| 7.1 | Example Quality Control Output for file T1.nii.gz | 28 |
| 7.2 | Example Quality Control Output for file BOLD.nii.gz | 31 |
| 7.3 | General Analytical Flow Chart | 36 |

# 1 Document preamble

| | |
|---|---|
| **Study** | MSB-C002 |
| **Client Contact** | Simone Grimm, simone.grimm@charite.de |
| **SBG Contact** | Maarten Mennes, analysis@sbgneuro.com |
| **Revision Date** | 23 March 2022 |
| **Version** | 1.0 - final |

| **Prepared by:** | **Submitted to:** |
|---|---|
| **SBGneuro Limited**<br><br>John Eccles House<br>Science Park<br>Robert Robinson Ave<br>Littlemore<br>Oxford, OX4 4GP<br>United Kingdom<br><br>Registered in England & Wales (No. 08183417) | Charité Research Organisation GmbH<br><br>Charitéplatz 1<br><br>10117 Berlin<br><br>Germany |

Copyright © 2022 SBGneuro Ltd. All rights reserved.

The material presented in this document and contained herein is proprietary and confidential to SBGneuro Ltd. and is intended for SBGneuro employees and its clients use only.

Material provided herein is confidential property and copyright protected by SBGneuro Limited. No part of said material can be reproduced or transmitted in any form or by any means, electronic or mechanical, including photocopy, recordings, emailing, or any information storage and retrieval system now or to be invented, without permission in written form from:

**SBGneuro Limited**

**John Eccles House**
**Science Park**
**Robert Robinson Ave**
**Littlemore**
**Oxford, OX4 4GP**
**United Kingdom**

**Registered in England & Wales (No. 08183417)**

# 2 Document revision history

| Version | Revision Details | Revision date | Revisions made by |
|---|---|---|---|
| 1.0 - final | Initial full analysis plan for IRB review | 23 March 2022 | Maarten Mennes<br><br>Christian Beckmann |
| | | | |
| | | | |
| | | | |

# 3 Approval

| | | |
|---|---|---|
| **SBG Author** | | |
| **Dr. Maarten Mennes (Head of MR Analysis)** | **Signature** | **Date (dd-Mmm-yyyy)** |
| **SBG Reviewer / Approval** | | |
| **Dr. Christian Beckmann (Director)** | **Signature** | **Date (dd-Mmm-yyyy)** |
| **Sponsor Approval** | | |
| **Dr. Simone Grimm** | **Signature** | **Date (dd-Mmm-yyyy)** |

# 4 Executive Summary

This Statistical Analysis Plan (SAP) summarizes the MRI analysis methodology and defines the processes, roles, and responsibilities of Medical School Berlin and SBGneuro Ltd. in the context of analyses for study MSB-C002. The analyses of MRI data received by SBGneuro Ltd. will be conducted under oversight by Dr. Christian Beckmann. The purpose of this SAP is to describe how the data received in the context of the MSB-C002 study will be quality controlled and analyzed by SBGneuro Ltd.

The included analyses and analysis methodology in this SAP is based on current knowledge. A review of the included analyses and applicable methodology will be performed following completion of all individual subject and group analyses to make any necessary adaptations. If adaptations are necessary, the SAP will be updated at that time. Exploratory MRI analysis results will not be used in support of study inclusion or exclusion criteria or main study endpoints.

## 4.1 Introduction

## 4.2 Study Design

MSB-C002 is a double blind, placebo-controlled, randomized, single dose, parallel-group design to assess the effects of a single dose of amisulpride on BOLD (blood oxygenation level dependent) responses during reward- and motivation-related processing using functional Magnetic Resonance Imaging (fMRI) in healthy volunteers (HV) and patients with Major Depressive Disorder (MDD). This SAP describes analyses performed on the task-based fMRI and the arterial spin labelling data.
The study includes acquisition of the following imaging modalities that can be used for endpoint derivation:

- T1-weighted structural image (T1)
- task-based functional Magnetic Resonance Imaging (fMRI)
- arterial spin labelling (ASL) to examine blood flow
- resting state functional Magnetic Resonance Imaging (rs-fMRI)

## 4.3 Trial Objectives and Endpoints

The objectives of the MSB-C002 study are to assess the effects of a single dose of amisulpride on functional brain changes induced by reward- and motivation-related cognitive challenge using functional Magnetic Resonance Imaging (fMRI) in HV and MDD to test whether amisulpride increases brain activation associated with reward- and motivation-related processing in MDD, but not in HV.

© SBGneuro Ltd

The primary endpoints of efficacy are the functional brain changes (expressed as %BOLD signal change for the contrasts of interest) induced by the Monetary Incentive Delay task (MID) under amisulpride compared to placebo in both the HV and MDD population.

## 4.4 Blinding and Unblinding

All analyses described in this SAP will be carried out on data that is fully blinded for dosage and diagnosis. In case of group analyses SBGneuro Ltd. may be partially unblinded in accordance to the needs of the specific analyses that are planned.

## 4.5 Image Acquisition

Table 1 specifies the imaging modalities that will be collected during the imaging visits specified for this study. Further details regarding the acquisition will be available in the **Imaging Manual** that will be made available for this study.

Table 1: Scheduled On-Protocol Subject Imaging

| Imaging Visits/Periods | Schedule | Imaging Data |
|---|---|---|
| **Visit 3 - Study Day 01** | post administration | • T1w structural imaging<br>• rs-fMRI<br>• MID task fMRI<br>• Instrumental Learning task fMRI<br>• Arterial Spin Labelling (ASL) |
| **Visit 4 - Study Day 8 +- 2** | post administration | • T1w structural imaging<br>• SID task fMRI<br>• Effort-Based Decision-Making task fMRI |

## 4.6 Endpoints and reporting

For the MRI aspect of this study endpoints will been defined, and are to be reported on for every collected session, if the data allows. The endpoints, the way in which they are reported, and the reporting frequency are further specified in a **Data Transfer Specification**.

© SBGneuro Ltd

Not controlled when exported
exported on March 23 2022

# 5 MRI Analysis Design and Methodology

## 5.1 Schedule for MRI Analysis

MRI scans will be analyzed on an ongoing basis as they are received. MRI group analysis can take place across all participants providing all available subject MRI scans have been received and analyzed.

## 5.2 Data for MRI Analysis

MRI data in DICOM format will be sent by the imaging site to SBGneuro Ltd. via secure ftp transfer.

Applicable subject information will be sent by the imaging site to SBGneuro Ltd. via comma separated files (.csv) files.

MRI data received by SBGneuro Ltd. as part of the transfer, but not described in this analysis plan will be ignored. Such data will not be processed in any way unless specifically requested, triggering a revision of this analysis plan. SBGneuro Ltd. will inquire with Medical School Berlin in case unexpected data are received.

## 5.3 Handling Missing Data

Every attempt will be made to avoid missing data. All participants will be included in the analyses, as per the analysis set, using all non-missing data available. SBGneuro Ltd. will not use imputation processes to estimate missing data.

## 5.4 MRI Quality Control

### 5.4.1 Data Completeness and Identifying Information

After receipt of a dataset (as outlined in the SBGneuro Upload Guidance that is provided to the client contact) SBGneuro Ltd. will check the provided data archive for completeness of the included imaging files. Data archives will be checked against the expected scans. Any data that is included in the archive and falls outside of the expected images to be uploaded will be ignored for further analyses. SBGneuro Ltd. will verify with the client or imaging site what the purpose of the extra scans is, and an appropriate course of action will be determined. In case of missing data SBGneuro Ltd. will verify with the client whether the missing data is in accordance with expectations (e.g., uncompleted or aborted scans).

© SBGneuro Ltd

Not controlled when exported
exported on March 23 2022

Unless otherwise agreed upon it is the client's responsibility to remove participant identifying information as required from the DICOM image headers. In case DICOM data are provided as part of uploaded data, SBGneuro Ltd. will check the DICOM header files for potentially remaining participant identifying information. To this end we will check all non-proprietary DICOM header fields. In case any identifying information is found SBGneuro Ltd. will consult with the client to determine the appropriate course of action in accordance with the client's and project's regulations regarding processing of such information. Required actions could include deletion of the data from the SBGneuro Ltd. compute infrastructure. In such case, affected data will be deleted from any back-up or archive that SBGneuro Ltd. might have made in the time between acceptance of the data and the retrieval of identifying information. In case uploaded data contains NIFTI imaging files only these checks are deemed unnecessary as participant identifying information cannot be stored in the NIFTI header.

### 5.4.2 Data Quality

All MR images will be subjected to visual and quantitative quality control.

- Image headers are checked for correspondence of the imaging parameters with the study sequences. Critical inconsistencies will be flagged and the imaging site will be contacted to resolve any unplanned changes to sequence parameters.
- Structural images will be inspected for image quality. During this proces images are not read by a qualified radiologist, yet SBGneuro Ltd. image analysts are instructed to flag potential incidental findings in the form of aberrant brain anatomy. In this case SBGneuro Ltd. will contact the imaging site and suggest further investigation according to sponsor's procedures regarding incidental findings. SBGneuro Ltd. cannot be held responsible for not flagging incidental abnormal brain anatomy.
- Other image modalities will be visually inspected for gross imaging artifacts and usability for endpoint derivation will be assessed. Where possible quantitative metrics will be used to decide on the suggested inclusion or exclusion of images from further analysis.

### 5.4.3 Modality-specific QC fail criteria

**T1-weighted structural images** will be marked as QC FAIL when:

- excessive head motion renders the image unusable for further registration purposes (non-quantifiable)
- the bounding box is improperly placed, leaving part of the brain outside the bounding box
- image quality prevents reliable extraction of the planned endpoints (non-quantifiable)

- an incidental finding is found that would impact image analysis or derived endpoints (non-quantifiable)

Note that a T1 QC fail could impact the derivation of endpoints from the other modalities. This is the case when a T1 image is deemed unusable for registration purposes, which will prevent deriving any endpoints in MNI152 standard space for that participant or session. It will also prevent inclusion of the participant or session in whole-brain group-level analyses. This can be circumvented in the case of multi-session data, where one of the other sessions of the same participant has an T1 of acceptable quality, as this is not expected to change significantly between sessions.

**rs- and task-fMRI** data will be marked as QC FAIL when:

- any absolute head movement exceeds 4mm
- the total scan length is significantly reduced, because of an aborted scan
- whole-brain average tSNR is below 2 standard deviations of the study's population mean. This is only quantifiable after analysis of all data.
- the bounding box is improperly placed, leaving parts of the brain needed for endpoint extraction outside the bounding box (non-quantifiable)
- general image quality prevents reliable extraction of the planned endpoints (non-quantifiable)
- anatomical abnormalities prevents reliable extraction of the planned endpoints (non-quantifiable)
- image artifacts or incorrect image reconstructions are observed that would prevent derivation of planned endpoints (non-quantifiable)
- an incidental finding is found that would impact image analysis or derived endpoints (non-quantifiable)

**ASL** data will be marked as QC FAIL when:

- excessive head motion renders the image unusable for endpoint extraction (non-quantifiable)
- the bounding box is improperly placed, leaving parts of the brain needed for endpoint extraction outside the bounding box (non-quantifiable)
- image quality prevents reliable extraction of the planned endpoints (non-quantifiable)
- not all labelling scans are available, preventing calculation of blood flow
- an incidental finding is found that would impact image analysis or derived endpoints (non-quantifiable)

© SBGneuro Ltd

Not controlled when exported
exported on March 23 2022

### 5.4.4 Analysis Inclusion and Exclusion

Endpoints will be derived from every dataset if the dataset allows deriving endpoints. Suggested inclusion/exclusion of provided endpoint data based on data quality will be provided as part of the data transfer as specified in the **Data Transfer Specification**.

### 5.4.5 Reporting on initial QC checks

In case of aberrant findings SBGneuro Ltd. will notify the client within **5** working days after acceptance of the data archive.

## 5.5 MRI Analysis

### 5.5.1 Analysis Software

All MRI data (pre)processing and analysis will be performed using FMRIB's Software Library (FSL[1]; licensed for commercial use to SBGneuro Ltd) and in-house created analyses scripts and pipelines using the *bash* or *Python* programming languages.

### 5.5.2 T1-weighted structural analysis

Registration

T1-weighted anatomical images will be used to bring the fMRI and ASL data to MNI152 anatomical standard space which will allow deriving anatomically correctly referenced endpoints from pre-specified locations in the human brain. MNI152 standard space is an anatomical reference space that is commonly used across the neuroimaging domain. Deriving endpoints in accordance to this reference space will allow comparison of results with results from already available neuroimaging studies.

T1 data will be aligned to MNI152 standard space using linear alignment via *FSL FLIRT* with 12 degrees of freedom[2]. Registration will subsequently be refined using non-linear steps as implemented in *FSL FNIRT*. We will also obtain the inverse of the the resulting transformations which will allow bringing region masks from MNI152 space to the participant's native space.

This study does not include endpoints derived from the T1 images. T1 images are solely used to facilitate analyses of the other imaging modalities included in the current study. Here, the T1 images will be used for registration purposes as described above. In addition, we will use FSL FIRST[3] to derive participant-

level segmentations of subcortical brainstructures, including the ventral striatum. These regions will be used as regions of interest for endpoint extraction in the other imaging modalities.

### 5.5.3 Task-based fMRI analyses

#### Preprocessing, 1st-level analyses and Endpoint extraction

*Preprocessing*

Task-based fMRI data allow investigating changes in oxygen levels of the blood in the brain via the Blood Oxygenation Level Dependent (BOLD) response. Blood oxygen levels are considered a proxy for neuronal activity, as the human body responds to an increase in neuronal activity in a brain region by increasing the influx of oxygen rich blood in oder to supply the active neurons with needed nutrients. By indexing changes in the brain's oxygen consumption while performing specific cognitive operations (e.g., reacting to a stimulus) we can assess which regions in the brain preferentially react to the stimuli in question.

Task fMRI data will first be realigned to correct for participant head motion. Where available fieldmap scans are used to correct for EPI distortions, and a 5mm FWHM spatial smoothing will be applied to the data. ICA components will be extracted and ICA-AROMA [4] will be applied to identify and remove secondary effect of head motion. Finally, a temporal 0.01Hz high-pass filter will be applied to remove any scanner drifts. We will obtain a transformation from the fMRI data to the participant's high resolution T1 anatomical space using *FSL BBR* (Boundary Based Registration). The obtained transformation can be combined with the T1 to MNI152 registration to bring fMRI BOLD data from the participant's native space to the MNI152 standard space.

*1st-level Analyses*

The preprocessed data will be used in participant-level 1st-level analyses used to obtain the contrasts of interest. This happens within the context of the General Linear Model (GLM) as implemented in *FSL FEAT*. For each task specific regressors are constructed and entered into the GLM yielding participant-level activation maps for contrasts of interest between certain regressors (see task-specifics below). For each task, onset times are obtained from the log files that are provided for each fMRI dataset. The participant-level activation maps are expressed in terms of Z-statistic scores and percent BOLD signal change for the specified contrast.

© SBGneuro Ltd

*Endpoints*

For each task we will report statistics for specific contrasts obtained for each participant. Contrasts that are reported on are specified in the **Data Transfer Specification**. Both Z-statistic scores and percent signal change will be summarized in regions of interest (ROI) and be reported as the median and average within the region, as well as the 90th percentile across scores obtained within the region. ROI are further specified in the **Data Transfer Specification**.

### MID and SID tasks

The Monetary Incentive Delay (MID) task assesses reward anticipation and receipt. Participants are presented with a cue indicating subsequent gain or no gain of money when presented with a target to which participants have to respond as quickly as possible. Correct responses are referred to as hits, too fast/slow or missed responses are called misses. The Social Incentive Delay (SID) task is equivalent to the MID task, but instead of a monetary reward participants are presented with a social reward in the form of happy faces. An overview of the task design and example stimuli are shown in Figure 1. There are three levels of reward. The main contrast of interest is *CueHighGain* > *CueNoGain*, contrasting cues indicating that the largest reward could be obtained versus cues indicating that no reward can be obtained in the ensuing trial.

### Instrumental Learning Task

The Instrumental Learning Task (ILT) assesses learning through reward. Participants are presented with non-descript cues (e.g., from a sign font) where one cue within a pair is associated with a (probabilistic) reward upon selection. Gain trials (where an actual reward is obtained) are mixed with Neutral trials (where no reward is received). Example stimuli are shown in Figure 2. The contrasts of interest are a comparison of the Gain > Neutral trials in the cue and the feedback period. These are modelled and obtained separately, i.e., CueGain > CueNeutral and FeedbackGain > FeedbackNeutral.

### Effort-based Decision Making Task

The Effort-based Decision Making Task measures neural responses to required effort and magnitude of reward. Participants can receive rewards related to the amount of grip strength they can exert on a hand-grip-response-device. On each trial participants a presented with a choice between performing no effort for a small reward or a high(er) effort for a high(er) reward. Example stimuli are shown in Figure 3. The

contrasts of interest compare the CueHighReward > CueLowReward and the CueHighEffort > CueLowEffort.

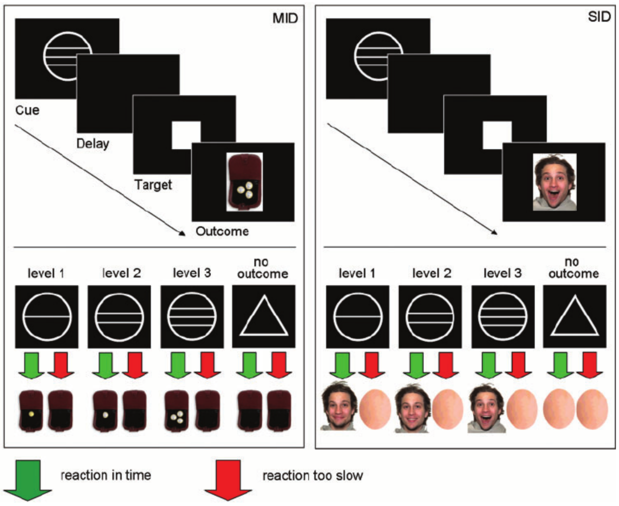


**Figure 1: Example stimuli for MID and SID tasks.**

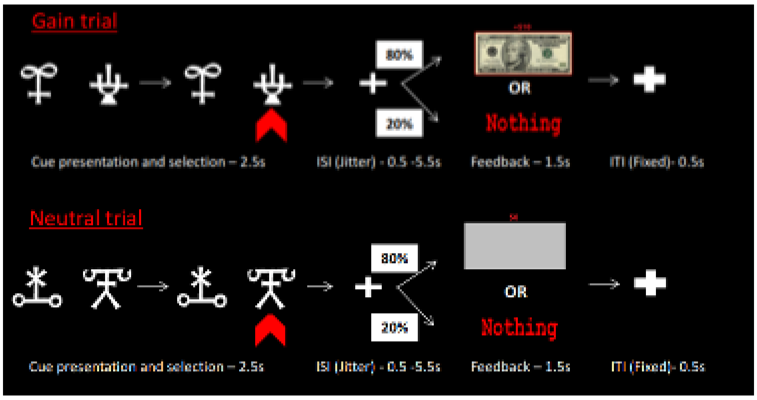


**Figure 2: Example Gain and Neutral trials from the Instrumental Learning Task.**

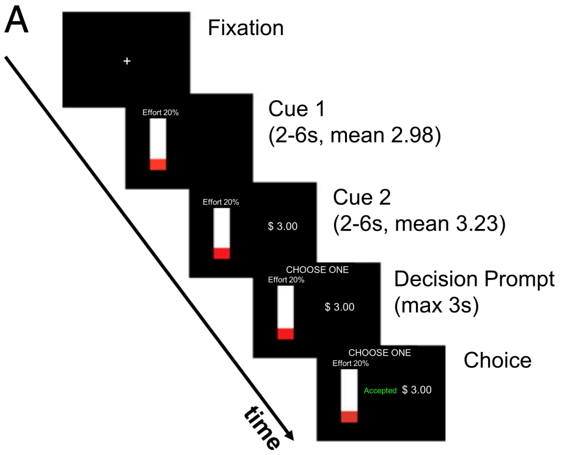


**Figure 3: Example trial for the Effort-based Decision Making task.**

### 5.5.4 Resting state fMRI analyses

Resting state fMRI data will be preprocessed similar to the task-based fMRI data. In addition to the preprocessing described above signal from participant-level cerebra-spinal fluid and white matter masks will be extracted and regressed out of the pre-processed data to exclude spurious correlations related to signal from these regions.

After preprocessing time series will be extracted from key ROIs via extraction of the first Principal Component extracted across the signal from all voxels within the ROI. Temporal correlation of the extracted time series is a measure of inter-regional functional connectivity. Fisher-Z transformed correlation values between a-priori specified ROIs are reported as endpoints for further statistical assessment in light of the planned group comparisons.

### 5.5.5 ASL analysis

ASL is a non-invasive technique in which blood water is magnetically “labeled” in the neck. Next, a post-labeling delay is introduced to allow the labeled blood to travel to the brain tissue and then an image of the brain is acquired. An additional image is obtained in which the blood water is not labeled, and when the two images are subtracted all of the tissue signal is removed, leaving a map of where the labeled

blood has accumulated. In addition, we can acquire data at multiple post-labeling delays and fit the resulting signals to a physiological model. After signal calibration, this allows to extract quantitative estimates of cerebral blood flow, accounting for differences in blood arrival time across the brain.

For the current study we take data acquired at a range of post-labeling delays and two calibration scans acquired with phase-encoding in opposite directions and correct them for subject motion. The calibration images with opposed phase-encoding direction are used to estimate distortions caused by magnetic field inhomogeneity, and these corrections are then applied to all the ASL data. A correction for receive coil non-uniformity is applied, to prevent bias in the resulting blood flow estimates. The label and control images are subtracted to generate perfusion images at each post-labeling delay, which are then fit to a well-established physiological model. This model can separate signals within large arteries from tissue perfusion signal to prevent bias in the resulting cerebral blood flow estimates. A spatial prior on the cerebral blood flow values is used to regularise the fitting procedure without directly smoothing the data. The resulting parameter estimates are registered on to the T1-weighted structural image acquired in the same participant. Signal calibration is achieved through automatic generation of a ventricle mask registered from MNI152 standard space to the participant's structural image and then on to the ASL calibration data. This is used to estimate the equilibrium magnetisation of cerebrospinal fluid, which can be converted into a calibration factor to allow the cerebral blood flow estimates to be expressed in absolute physiological units (ml/100g/min), accounting for the specific scan parameters used in the study.

ASL Endpoints

For each ASL dataset we will report the absolute cerebral blood flow within the left and right amygdala, left and right hippocampus, left and right anterior insula, left and right dorso-lateral prefrontal cortex (DLPFC), and left and right anterior cingulate cortex (ACC). We will also provide these values normalised by the mean cerebral blood flow within the participant's grey matter, which can help to account for subject-to-subject differences in physiology, such as those arising from factors such as age and caffeine intake.

### 5.5.6 ROI definition

As described above endpoints will be extracted from a set of regions of interest (ROIs). ROI are further specified in the **Data Transfer Specification** and for this study include:

- left and right ventral striatum, including caudate, putamen and nucleus accumbens.
- left and right ventral tegmental area

- left and right ventral palladium
- dorsal anterior cingulate cortex
- ventromedial prefrontal cortex, orbitofrontal cortex
- For resting state fMRI analyses we will include: Default Mode Network (posterior cingulate, vmPFC and medial temporal lobe), Salience Network (amygdala, insula and dorsal anterior cingulate), Central Executive Network (dorsolateral prefrontal cortex, premotor cortex, precuneus)
- ROIs are defined based on available anatomical atlases or derived from key publications using the described fMRI tasks. ROIs will be defined a-priori and fully blinded as described in the protocol.
- ROI masks are defined in MNI152 standard space and transformed from MNI152 standard space to each subject's native fMRI or ASL space. Transformations between each subject's native space and MNI152 standard space were obtained as described under *Registration* (see above).

© SBGneuro Ltd

Not controlled when exported
exported on March 23 2022

# 6 References

1. [1] Jenkinson, M., Beckmann, C. F., Behrens, T. E. J., Woolrich, M. W., & Smith, S. M. (2012). FSL. *NeuroImage*, *62*(2), 782–790. https://doi.org/10.1016/j.neuroimage.2011.09.015
2. [1] Jenkinson, M., Bannister, P., Brady, M., & Smith, S. (2002). Improved Optimization for the Robust and Accurate Linear Registration and Motion Correction of Brain Images. *NeuroImage*, *17*(2), 825–841. https://doi.org/10.1006/nimg.2002.1132
3. [1] Patenaude, B., Smith, S. M., Kennedy, D. N., & Jenkinson, M. (2011). A Bayesian model of shape and appearance for subcortical brain segmentation. *NeuroImage*, *56*(3), 907–922. https://doi.org/10.1016/j.neuroimage.2011.02.046
4. [1] Pruim et al. 2015. ICA-AROMA: A robust ICA-based strategy for removing motion artifacts from fMRI data. *NeuroImage*. 112, 267–277.

# 7 Appendix

## 7.1 Example Quality Control Output for file T1.nii.gz

### 7.1.1 Raw data:

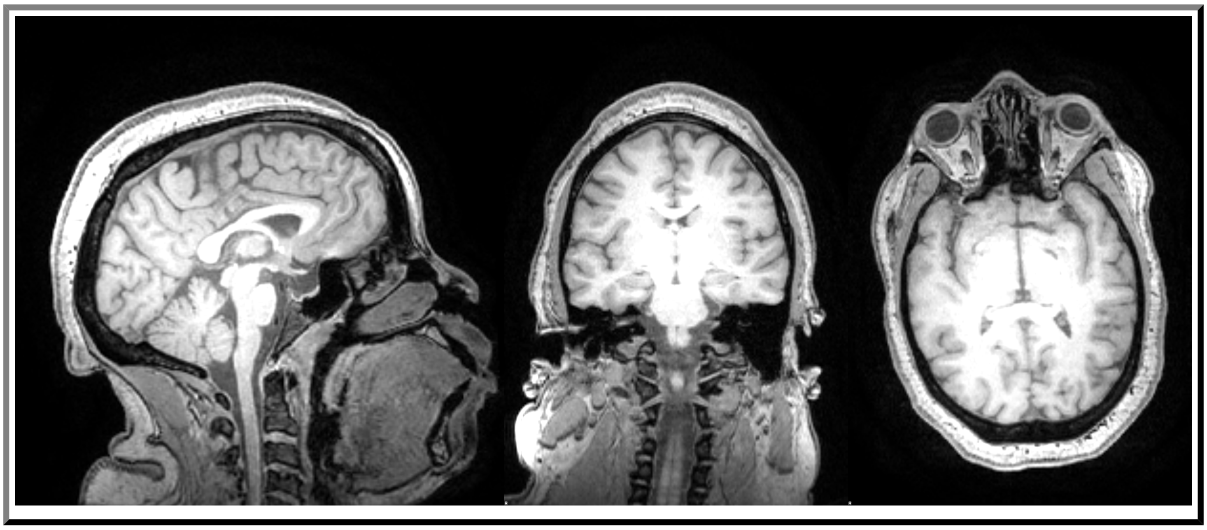

### 7.1.2 Phase 1: Header Check

| | |
|---|---|
| **Parameter_Name:** | X_Dimension |
| **Test_Passed:** | False |
| **Actual_Value:** | 180.0 |
| **Protocol_Value:** | 170 |

| | |
|---|---|
| **Parameter_Name:** | Y_Dimension |
| **Test_Passed:** | True |
| **Actual_Value:** | 256.0 |
| **Protocol_Value:** | 256 |

| | |
|---|---|
| **Parameter_Name:** | Z_Dimension |
| **Test_Passed:** | True |

| | |
|---|---|
| **Actual_Value:** | 256.0 |
| **Protocol_Value:** | 256 |

| | |
|---|---|
| **Parameter_Name:** | Temporal_Dimension |
| **Test_Passed:** | True |
| **Actual_Value:** | 1.0 |
| **Protocol_Value:** | 1 |

| | |
|---|---|
| **Parameter_Name:** | X_FOV |
| **Test_Passed:** | False |
| **Actual_Value:** | 180.0 |
| **Protocol_Value:** | 204 - 204.1 |

| | |
|---|---|
| **Parameter_Name:** | Y_FOV |
| **Test_Passed:** | True |
| **Actual_Value:** | 256.0 |
| **Protocol_Value:** | 256 |

| | |
|---|---|
| **Parameter_Name:** | Z_FOV |
| **Test_Passed:** | True |
| **Actual_Value:** | 256.0 |
| **Protocol_Value:** | 256 |

| | |
|---|---|
| **Parameter_Name:** | Phase1_sizes_Isometry |
| **Isometry_Expected:** | Yes |
| **Protocol_Value:** | 1 |
| **Test_Passed:** | True |

| | |
|---|---|
| **Parameter_Name:** | X_Pixel_Size |
| **Test_Passed:** | False |
| **Actual_Value:** | 1.0 |
| **Protocol_Value:** | 1.2 - 1.21 |

| | |
|---|---|
| **Parameter_Name:** | Y_Pixel_Size |
| **Test_Passed:** | True |
| **Actual_Value:** | 1.0 |
| **Protocol_Value:** | 1 |

| | |
|---|---|
| **Parameter_Name:** | Z_Pixel_Size |
| **Test_Passed:** | True |
| **Actual_Value:** | 1.0 |

| | |
|---|---|
| **Protocol_Value:** | 1 |

| | |
|---|---|
| **Parameter_Name:** | Temporal_Size |
| **Test_Passed:** | False |
| **Actual_Value:** | 0.00695999991149 |
| **Protocol_Value:** | 1 |

### 7.1.3 Phase 2: Registration Check

Registration to atlas

### 7.1.4 Phase 3: Data check

| | |
|---|---|
| **Test_Name:** | Spatial_SNR |
| **Test_Passed:** | True |
| **Actual_Value:** | 1024.36802174 |
| **Value_Limits:** | 1.0 < x < inf |

| | |
|---|---|
| **Test_Name:** | Spatial_Contrast |
| **Test_Passed:** | True |
| **Actual_Value:** | 335.50943073 |
| **Value_Limits:** | 1.0 < x < inf |

| | |
|---|---|
| **Test_Name:** | Spatial_Entropy |
| **Test_Passed:** | False |
| **Actual_Value:** | 0.870938 |
| **Value_Limits:** | -inf < x < 0.86 |

### 7.1.5 Phase 4: ICA-based checks

### 7.1.6 Phase 5: Motion-based checks

## 7.2 Example Quality Control Output for file BOLD.nii.gz

### 7.2.1 Raw data:

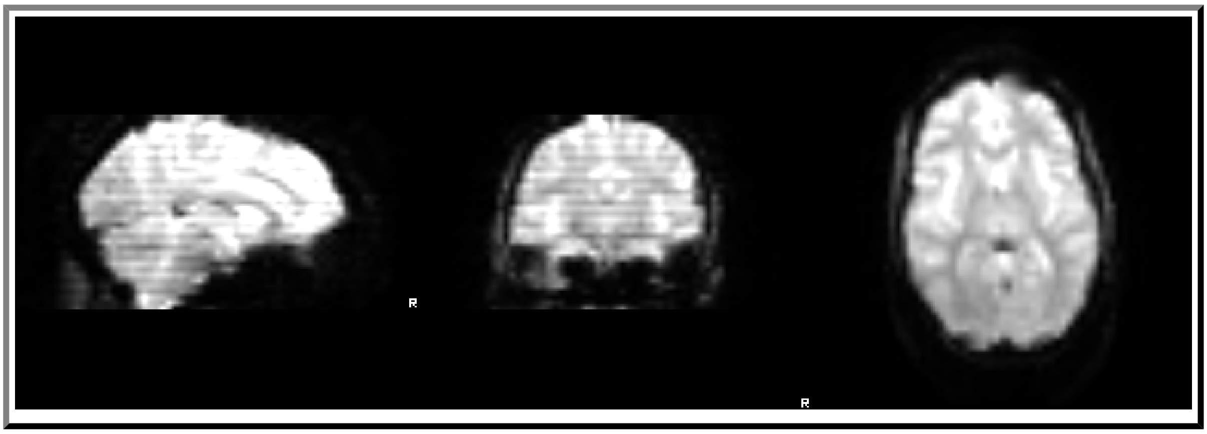

### 7.2.2 Phase 1: Header Check

| | |
|---|---|
| **Parameter_Name:** | X_Dimension |
| **Test_Passed:** | True |
| **Actual_Value:** | 64.0 |
| **Protocol_Value:** | 64 |

| | |
|---|---|
| **Parameter_Name:** | Y_Dimension |
| **Test_Passed:** | True |
| **Actual_Value:** | 64.0 |
| **Protocol_Value:** | 64 |

| | |
|---|---|
| **Parameter_Name:** | Z_Dimension |
| **Test_Passed:** | True |
| **Actual_Value:** | 35.0 |
| **Protocol_Value:** | 35 |

| | |
|---|---|
| **Parameter_Name:** | Temporal_Dimension |

| | |
|---|---|
| **Test_Passed:** | False |
| **Actual_Value:** | 150.0 |
| **Protocol_Value:** | 240 |

| | |
|---|---|
| **Parameter_Name:** | X_FOV |
| **Test_Passed:** | True |
| **Actual_Value:** | 240.0 |
| **Protocol_Value:** | 239.9 - 240.1 |

| | |
|---|---|
| **Parameter_Name:** | Y_FOV |
| **Test_Passed:** | False |
| **Actual_Value:** | 240.0 |
| **Protocol_Value:** | 229.9 - 230 |

| | |
|---|---|
| **Parameter_Name:** | Z_FOV |
| **Test_Passed:** | True |
| **Actual_Value:** | 119.00455 |
| **Protocol_Value:** | 118.9 - 119.1 |

| | |
|---|---|
| **Parameter_Name:** | Phase1_sizes_Isometry |
| **Isometry_Expected:** | No |
| **Protocol_Value:** | 1 |
| **Test_Passed:** | True |

| | |
|---|---|
| **Parameter_Name:** | X_Pixel_Size |
| **Test_Passed:** | True |
| **Actual_Value:** | 3.75 |
| **Protocol_Value:** | 3.74 - 3.76 |

| | |
|---|---|
| **Parameter_Name:** | Y_Pixel_Size |
| **Test_Passed:** | True |
| **Actual_Value:** | 3.75 |
| **Protocol_Value:** | 3.74 - 3.76 |

| | |
|---|---|
| **Parameter_Name:** | Z_Pixel_Size |
| **Test_Passed:** | True |
| **Actual_Value:** | 3.40013432503 |
| **Protocol_Value:** | 3.39 - 3.41 |

| | |
|---|---|
| **Parameter_Name:** | Temporal_Size |
| **Test_Passed:** | True |

© SBGneuro Ltd

Not controlled when exported
exported on March 23 2022

| | |
|---|---|
| **Actual_Value:** | 2.0 |
| **Protocol_Value:** | 1.99 - 2.01 |

### 7.2.3 Phase 2: Registration Check

Registration to atlas

| | |
|---|---|
| **Test_Name:** | Scale1 |
| **Test_Passed:** | True |
| **Actual_Value:** | 1.214749 |
| **Value_Limits:** | 0.7 < x < 1.3 |

| | |
|---|---|
| **Test_Name:** | Scale2 |
| **Test_Passed:** | True |
| **Actual_Value:** | 1.096126 |
| **Value_Limits:** | 0.7 < x < 1.3 |

| | |
|---|---|
| **Test_Name:** | Scale3 |
| **Test_Passed:** | True |
| **Actual_Value:** | 1.268912 |
| **Value_Limits:** | 0.7 < x < 1.3 |

| | |
|---|---|
| **Test_Name:** | Shear1 |
| **Test_Passed:** | False |
| **Actual_Value:** | 0.021672 |
| **Value_Limits:** | -0.02 < x < 0.02 |

| | |
|---|---|
| **Test_Name:** | Shear2 |
| **Test_Passed:** | True |
| **Actual_Value:** | -0.016461 |
| **Value_Limits:** | -0.02 < x < 0.02 |

| | |
|---|---|
| **Test_Name:** | Shear3 |
| **Test_Passed:** | False |
| **Actual_Value:** | 0.067178 |
| **Value_Limits:** | -0.02 < x < 0.02 |

| | |
|---|---|
| **Test_Name:** | RegistrationVolumeProportion |
| **Test_Passed:** | True |
| **Actual_Value:** | 0.635371526091 |

| | |
|---|---|
| **Value_Limits:** | 0.6 < x < 1.2 |

| | |
|---|---|
| **Test_Name:** | Dice |
| **Test_Passed:** | True |
| **Actual_Value:** | 0.847078187761 |
| **Value_Limits:** | 0.7 < x < inf |

| | |
|---|---|
| **Test_Name:** | OverlappingProportion |
| **Test_Passed:** | True |
| **Actual_Value:** | 0.74865669042 |
| **Value_Limits:** | 0.7 < x < inf |

| | |
|---|---|
| **Test_Name:** | Flirt_Cost_Function |
| **Test_Passed:** | True |
| **Actual_Value:** | 0.191378 |
| **Value_Limits:** | -inf < x < 0.5 |

### 7.2.4 Phase 3: Data check

| | |
|---|---|
| **Test_Name:** | Spatial_SNR |
| **Test_Passed:** | True |
| **Actual_Value:** | 1846.13457705 |
| **Value_Limits:** | 1.0 < x < inf |

| | |
|---|---|
| **Test_Name:** | Spatial_Contrast |
| **Test_Passed:** | True |
| **Actual_Value:** | 504.87062693 |
| **Value_Limits:** | 1.0 < x < inf |

| | |
|---|---|
| **Test_Name:** | Temporal_SNR |
| **Test_Passed:** | True |
| **Actual_Value:** | 5052.75396737 |
| **Value_Limits:** | 1.0 < x < inf |

| | |
|---|---|
| **Test_Name:** | Temporal_Contrast |
| **Test_Passed:** | True |
| **Actual_Value:** | 47.9676470017 |
| **Value_Limits:** | 1.0 < x < inf |

### 7.2.5 Phase 4: ICA-based checks

### 7.2.6 Phase 5: Motion-based checks

| | |
|---|---|
| **Test_Name:** | Bad_Motion_Vols |
| **Test_Passed:** | True |
| **Actual_Value:** | 0.12 |
| **Value_Limits:** | -inf < x < 0.4 |

| | |
|---|---|
| **Test_Name:** | DVARS |
| **Test_Passed:** | True |
| **Actual_Value:** | 17.1785342282 |
| **Value_Limits:** | -inf < x < 40.0 |

| | |
|---|---|
| **Test_Name:** | Mean_Motion_Rel |
| **Test_Passed:** | True |
| **Actual_Value:** | 0.0410019 |
| **Value_Limits:** | -inf < x < 0.15 |

| | |
|---|---|
| **Test_Name:** | Mean_Motion_Abs |
| **Test_Passed:** | True |
| **Actual_Value:** | 0.149374 |
| **Value_Limits:** | -inf < x < 0.15 |

| | |
|---|---|
| **Test_Name:** | Max_Motion |
| **Test_Passed:** | True |
| **Actual_Value:** | 0.303667 |
| **Value_Limits:** | -inf < x < 4.0 |

## 7.3 General Analytical Flow Chart

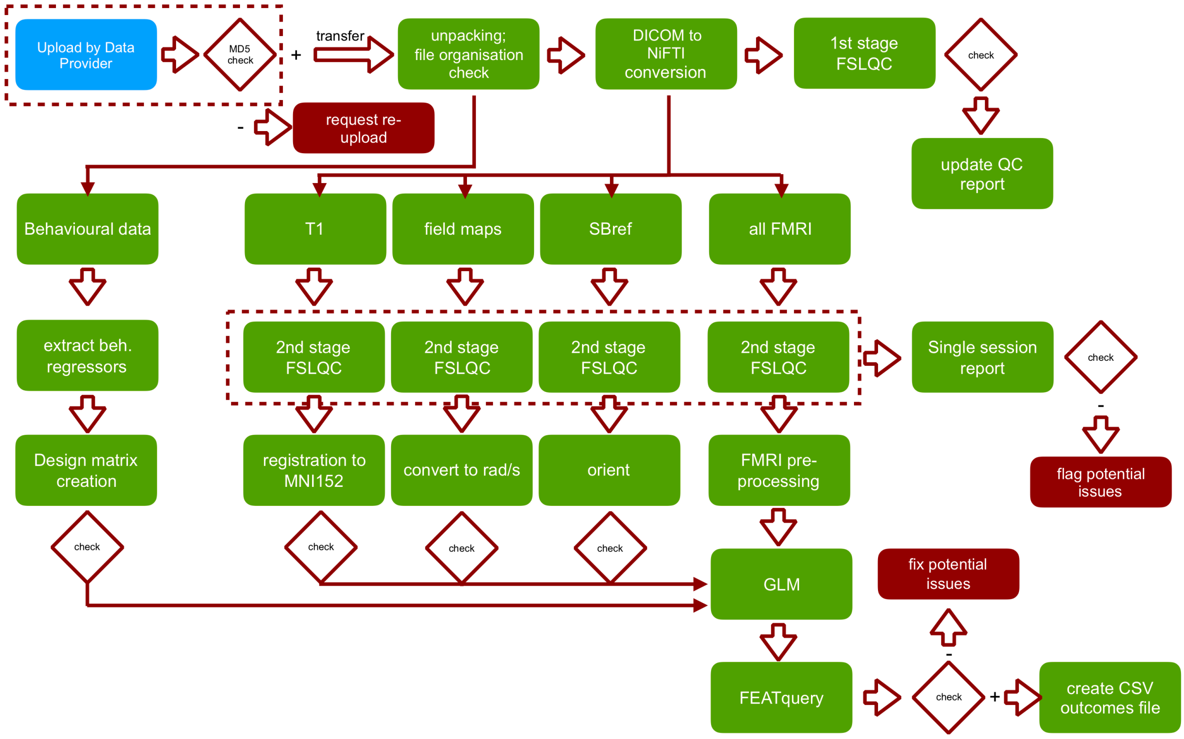


**Figure 4: Flow chart that illustrates the process involved in analysing MRI data. This chart serves as an example steps might be altered, added, or removed depending on the requirements specified in this SAP.**
